# Psychosocial Rehabilitation Training in the Treatment of Schizophrenia Outpatients: A Randomized, Psychosocial Rehabilitation training–and Monomedication-Controlled Study

**DOI:** 10.12669/pjms.292.2951

**Published:** 2013-04

**Authors:** Ling Wang, Jianchu Zhou, Xueqin Yu, Jihong Qiu, Bo Wang

**Affiliations:** 1 Ling Wang, Researcher, Research and Education Department, Chongqing Mental Health Center, China.; 2 Jianchu Zhou, Professor, Chief Researcher, Research and Education Department, Chongqing Mental Health Center, China.; 3 Xueqin Yu, Professor, Researcher, Research and Education Department, Chongqing Mental Health Center, China.; 4 Jihong Qiu, Associate Professor, Researcher, Out Patient Department, Chongqing Mental Health Center, China.; 5 Bo Wang, Professor, Researcher, Chongqing Mental Health Center, China.

**Keywords:** Schizophrenia, Psychosocial, Rehabilitation, Training, Relapse, Outcomes

## Abstract

***Objective:*** This study was designed to evaluate the efficacy of psychosocial rehabilitation intervention on schizophrenia.

***Methodology:*** One hundred forty schizophrenia outpatients in remission stage were randomized to either an antipsychotic monomedication (control group) or an antipsychotic monomedication plus a psychosocial rehabitation training (trial group). Positive and Negative syndrome Scale (PANSS), Disability Screening Schedule (SDSS) were performed longitudinally from baseline to month 18 to evaluate the efficacy.

***Results:*** Significant difference in relapse rate between the control group (42.9%) and the trial group (18.6%) was found at month 18. In patients who didn’t relapse, the trial group showed significantly lower PANSS and SDSS score (P<0.05) than did the control group after treatment.

***Conclusion:*** Psychosocial rehabilitation intervention could produce a better outcome in terms of reducing relapse and improving the social functioning in schizophrenia.

## INTRODUCTION

Schizophrenia is a kind of chronic mental disease characterized by high relapse rate and disability rate.^1^ According to Thara R and Suzuki Y, the relapse rate of schizophrenia ranges from 40% to 90%.^2^^,^^3^ In recent years, many influential studies, purposing to prevent relapse and improve social functioning for schizophrenia, have been conducted, but most of them have focused on efficacy of various drugs.^4^ Even with best practices, there are limitations to the effectiveness of medications in the treatment of schizophrenia.^5^ Although much previous findings have indicated that schizophrenia might do best with a combination of pharmacological and psychosocial intervention and many psychosocial interventions, including social skill training, family psychical education, illness management and recovery training, have been demonstrated effective in schizophrenia and other serious psychiatric illnesses treatment, but these treatments had no clear effects on relapse.^6^^-^^9^

Could clinical outcomes and social functioning of schizophrenia patients be further improved with a combination of an antipsychotic monomedication and a long-term systemic psychosocial interventions training?

This study, approved by Chongqing Mental Health Center (CMHC), was designed as a proof-of Concept study to determine whether an antipsychotic monomedication plus a Psychosocial Rehabitation Training (PsRT) would produce good clinical outcomes and enhance the social functioning at 18 months in Chinese schizophrenia population.

## METHODOLOGY

***Subject: ***Inclusion Criteria: Participants were screened by consultant psychiatrists(CMHC) to ensure that they met the following criteria: 1). ICD-10 and DSM-IV criteria for schizophrenia; 2). Criteria for clinical effectiveness of acute –phase treatment; 3).The decrease rate of the PANSS total score ≥50% or the PANSS total score ≤60 after acute –phase treatment; 4). Being consistent to a monomedication therapy; 5). Disease course was less than 5 years; 6). aged 16-50; 7). Being accompanied by at least one guardian for two years.

***Exclusion Criteria:*** 1). Severe physical illnesses or substance use; 2). A relocation plane in two years; 3). Severe physical disability that would interfere with the follow-up or training; 4). those who must take a combination medication or a prolonged action medicine; 5). Those who might have complicated problems; 6). Other mental disorders, mental retardation, dementia or severe cognitive disorder; 7). Pregnancy or lactation or a pregnancy plane in two years.

***Methodology: ***A randomly-controlled-prospective-match design was used in this study. Patients, after signing the written informed consent, were randomly divided into a trial group or a control group by a coin toss method then another patients, matched for sex, age (±5 years), marital status, occupational status and the medicine used in maintenance treatment, was assigned to the other group (i.e. a patient taking risperidone got the reverse side, then he would be assigned to the trial group, and another patient using risperidone as well would be matched and assigned to the control group, and vice versa. The control group would be treated with an antipsychotic monomedication which was generally recommended by World Psychiatric Association^10^ and the trial group would be administrated with the same antipsychotic monomedication plus a psychosocial rehabilitation training which was operated by a group of trained psychiatrists under the tutorial manual.

The PsRT was composed of four parts: 1). Basic psychiatry knowledge learning, in which patients and their family members would be educated. The purpose of this part was to improve the self-diagnosis skill for psychotic symptoms and develop their self- management ability of medicine and help participants to realize the importance of maintenance treatment. 2). Independent living skill training,^11^ operated through homework mainly, included: self care, individual behavioral norms, shopping, entertaining, party, etc. In order to enhance patients’ initiative of self-service in this part, family members were required to fill in a health daily record under the guidance of a psychiatrist, then the psychiatrist would discuss the health problems found in the record with their family members. 3). Social skill and vocational rehabilitation training was a behavioral intervention for schizophrenia that focused on social functioning^12^^,^^13^ which was made up of the following parts: Interpersonal communication in which patients would be asked to use “I am ……” to make sentences; Games, such as “back-to-back drawing”, “name explanation”, “body touching”; and problem solving, in this part patients would be required to give different solutions to different questions after group discussion., such as “ What will you do, if you found the goods you bought from a supermarket has a quality problem?”, “What will you do, if your friend or colleague misunderstood you?”; 4). Cognitive intervention: With the purpose of exploring whether mood and behavior could be modified by beliefs, assumptions and other cognitive factors,^14^ we used group and individual psychological intervention to lead patients to make self-analysis and discover their own problems.

In order to achieve a better training efficacy, participants (including family members) in trial group was divided into many subgroups (N=8-10) which were administrated with PsRT once-monthly (2 hours each time, mean attendance rate=80%) for 18 times. Participants who were absent for more than twice were excluded. Efficacy, including relapse rate, was assessed with Positive and Negative Syndrome Scale (PANSS), Social Disability Screening Scale (SDSS) and Schizophrenia Cognition Rating Scale (SCRS) at baseline and every 3 to 6 months. Since assessments were conducted for many times in this study, we choose the middle and end assessments for the final statistical analysis.

***Relapse criteria:*** At least one of the following criteria was met: 1). Any item score≥5 or the sum of any two items score ≥4 of: Conceptual disorganization, Hallucination, Suspiciousness, Mannerisms and Posturing, Unusual thought content in PANSS; 2). More than 2 symptoms relapsed and lasted over one week; 3). Those whose occupation or daily life was disturbed by psychotic symptoms or violence behavior, such as self-harm, harm to others and destruction, etc.; 4). Significant suicide behavior; 5). Rehospitalization or the rehospitalization needed. 6). The patients dismissed due to psychotic symptoms. The relapse rate was analyzed by a group of trained psychiatrist (concordance rate r=0.76-0.92, Kappa=0.65-0.87, P<0.01).

***Statistical analysis: ***Enumeration data was analyzed with chi square test with t-test and variance analysis through SPSS 17.0.

## RESULTS

Total number of patients who completed the study were 140 (27 male and 43 female) for each group. Mean age: control group 26.79±6.99 versus trial group 26.27±6.81. Marital status: 24 married and 46 unmarried in control group versus 23 married and 47 unmarried in trial group. Length of Education: 11.00±17.98 months for control group versus 31.54±18.76 months for trial group. Illness duration: 26.50±17.98 months for control group versus 31.54±18.76 months for trial group. PANSS total score at baseline: 41.7±7.1 for control group versus 41.4±6.8 for trial group. SDSS total score at baseline: 4.57±3.37 for control group versus 3.51±3.09 for trial group. Medications used in this trial were: risperidone (N=20, mean dosage: 3.15±1.34mg), quetiapine (N=20, mean dosage: 445±131mg), clozapine (N=20, mean dosage: 218±112mg), sulpiride (N=20, mean dosage: 585±166mg), olanzapine (N=10, mean dosage: 10.05±2.84mg), chlorpromazine (N=30, mean dosage: 303±219mg), aripiprazole (N=20, mean dosage: 15.75±5.68mg). Equilibrium test was applied to all the factors listed above and no significant group difference was found.

***Clinical outcomes (*** Table-I ***): ***The patients who received monomedication plus the psychosocial rehabitation training showed significantly lower relapse rate (2.9%, 18.6%) after treatment than did the patients in control group (12.9%, 42.9%) at month 6 and 18 respectively (p<0.05), meanwhile, patients treated with the psychosocial rehabitation training also had a higher stable rate (p<0.01).

***PANSS and SDSS Score (*** Table-II ***): ***Significant differences in positive symptom, negative symptom and PANSS mean total score between the patients who didn’t meet the relapse criteria at month 18 in both the groups were found (P<0.05), but there was no significant difference in general psychopathological symptoms (P>0.05). SDSS mean score difference (t=2.305, P<0.05) was not significant for the stable patients in both two groups.

**Table-I:** Overall outcomes at month 6 and month 18

*Treatment group*	*Stable*		*Relapsed*		*Dropout*		*Schedule switch*	
	*N*	*%*	*N*	*%*	*N*	*%*	*N*	*%*
Month 6								
Control group	57	81.4	9	12.9	3	4.3	1	1.4
Trial group	67	95.7	2	2.9	0	0	1	1.4
Month 18								
Control group	30	42.9	30	42.9	6	8.6	4	5.7
Trial group	50	71.4	13	18.6	4	5.7	3	4.3

Chi square test: x²=8.26, P<0.05 at month 6; x²=12.26, P<0.01 at month18

**Table-II:** PANS scores at month 18. (x(-)*±* s

	*Control group(30)*	*Trial group(50)*	*t*	*P*
Positive symptom score	*7.83 ± 0,79*	*7.44 ± 0.70*	*2.308*	*0.024*
Negative symptom score	*9.10 ± 1.69*	*8.08 ± 1.22*	*3.119*	*0.003*
General psychopathological	19.27 *± 2.50*	*18.76 ± 2.21*	*0.944*	*0.348*
Total score	36.20 *± 4.13*	*34.30 ± 3.47*	*2.206*	*0.030*

## DISCUSSION

A great number of schizophrenia patients relapse during maintenance treatment which lead to the degeneration of social functioning gradually, so prevention of relapse, reducing disability rate and promoting the recovery of social functioning are both the focus point and difficulty for psychiatrists. Liberman and other psychiatrists have used “social and dependence living skill” to train chronic schizophrenia patients during rehabitation stage, which proved significant efficacy on social function.^15^^,^^16^ This randomized control clinical trial examined whether adding a long-term systemic psychosocial rehabitation training to a monomedication improved clinical outcomes for schizophrenia outpatients during maintenance treatment. Except for pilot studies in China, we are aware of no other published clinical trial of psychosocial rehabitation trainings for schizophrenia outpatients. After treatment, the patients who received a monomedicaiton plus the psychosocial rehabitation training outperformed the monomedicaiton therapy in terms of relapse, psychical symptoms, metal retardation and social functioning. The optimistic achievements of this study may be due to following reasons: 1). A close relationship between patients and doctors was build which could ensure the professional help or guide being delivered timely. 2). Through physical rehabitation training, patients and family members learned more knowledge and skills to deal with the early symptoms of relapse and side effects which could increase adherence. 3). With the support from family members, the social adaptability of patients was improved after training. 4). Patients in this trial were recruited not long after acute phase treatment and their disease course was relatively short, so it was easy for them to achieve a better outcome after psychosocial rehabitation training. However, one limitation should be noted, the dropout rates for the control group and the trial group were 18.6% and 5.7% respectively and the relapse in this population was unknown. We found 83.7% of the relapses were caused by over reduction of dosage, unordered administration or withdrawal, so how to strengthen the adherence still need be studied further.
